# PD-L1 expression in lung cancer and its correlation with driver mutations: a meta-analysis

**DOI:** 10.1038/s41598-017-10925-7

**Published:** 2017-08-31

**Authors:** Minghui Zhang, Guoliang Li, Yanbo Wang, Yan Wang, Shu Zhao, Pu Haihong, Hongli Zhao, Yan Wang

**Affiliations:** 1 Department of Medical Oncology, Harbin Medical University Cancer Hospital, Harbin, 150081 Heilongjiang China; 2 Department of General Surgery, The Second Affiliated Hospital of Harbin Medical University, Harbin, 150040 Heilongjiang China; 3 Department of Surgical Oncology, Harbin Medical University Cancer Hospital, Harbin, 150081 Heilongjiang China; 4 Department of Medical Oncology, Heilongjiang Provincial Hospital, Harbin, 150030 Heilongjiang China

## Abstract

Although many studies have addressed the prognostic value of programmed cell death-ligand 1 (PD-L1) expression in lung cancer, the results remain controversial. A systematic search of the PubMed, EMBASE, and Cochrane Library databases was performed to identify the correlation between PD-L1 expression and driver mutations and overall survival (OS). This meta-analysis enrolled a total of 11,444 patients for 47 studies, and the pooled results showed that increased PD-L1 expression was associated with poor prognosis (HR = 1.40, 95% CI: 1.19–1.65, *P* < 0.001). In subgroup analysis stratified according to histology types, the pooled results demonstrated that increased PD-L1 expression was an unfavorable prognostic factor for non-small cell lung cancer (NSCLC) (HR = 1.26, 95% CI: 1.05–1.52, *P* = 0.01) and pulmonary lymphoepithelioma-like carcinoma (LELC) (HR = 3.04, 95% CI: 1.19–7.77, *P* = 0.02), rather than small cell lung cancer (SCLC) (HR = 0.62, 95% CI: 0.27–1.39, *P* = 0.24). The pooled ORs indicated that PD-L1 expression was associated with gender, smoking status, histology, differentiation, tumour size, lymph nodal metastasis, TNM stage and EGFR mutation. However, PD-L1 expression was not correlated with ALK rearrangement and KRAS mutations.

## Introduction

Lung cancer, broadly divided into small cell lung cancer (SCLC) and non-small cell lung cancer (NSCLC), is deemed as the leading cause of cancer-related deaths in both the United States and China^1, 2^. More than 70% of patients are diagnosed with advanced disease, which are not amenable to curative therapy. Although much progress has recently been made for lung cancer such as low-dose spiral screening, minimally invasive techniques for diagnosis and treatment, advances in radiation therapy and molecularly targeted therapies, patients with lung cancer are still facing a relatively low 5-year survival rate, merely 17.4%^3^. Thus, immunotherapies have been considered as a very promising therapeutic strategy for different tumour types.

Programmed death 1 (PD-1), a member of the CD28 family, is a key immune checkpoint receptor expressing on the surface of the activated T, B and NK cells and plays a crucial role in tumour immune escape^4^. Programmed cell death ligand 1(PD-L1), the mainly ligand of PD-1, is upregulated in different types of tumours, including breast cancer^5^, NSCLC^6^, colorectal cancer^7^, gastric cancer^8^, testicular cancer^9^ and papillary thyroid cancer^10^. PD-L1 delivers negative costimulatory signals and binds PD-1 to reduce cellular immune responses by inducing T-cell apoptosis or exhaustion. Blocking the PD-1/PD-L1 pathway with monoclonal antibodies (MoAbs) is currently considered to be the most promising approach, offering durable activity and long-term survival outcomes^11^. Several meta-analyses have demonstrated that not only is PD-L1 expression associated with adverse clinical and pathologic features but an increased risk of death in many cancer types^12–15^. However, date regarding the prevalence and prognostic role of PD-L1 expression in NSCLC remains controversial, particularly in SCLC and other types of lung cancers.

NSCLC is a disease that is characterized by driver mutation-defined molecular subsets, and alterations in epidermal growth factor receptor (EGFR), anaplastic lymphoma kinase (ALK), and KRAS are major oncogenic drivers in NSCLC^16^. However, the relationship between major driver mutations and PD-L1 expression remains unclear. A recent study showed that oncogenic EGFR mutations directly up-regulated PD-L1 protein expression on the surface of cells in NSCLC, and exposure to gefitinib also lead to PD-L1 up-regulation^17^. Another study showed the upregulation expression of PD-L1 in NSCLC as a result of an EGFR mutation and ALK rearrangement via common downstream signalling pathways mediated by PI3K-AKT and by MEK-ERK^18^, implicating driver mutations in the regulation of the expression of immunosuppressive molecules.

We therefore conducted a comprehensive meta-analysis to investigate the significance of PD-L1 expression as a prognostic marker and to determine the relation of PD-L1 expression to clinicopathological features and driver mutations in lung cancer patients.

## Results

### Search results and characteristics of studies

**Figure 1:**
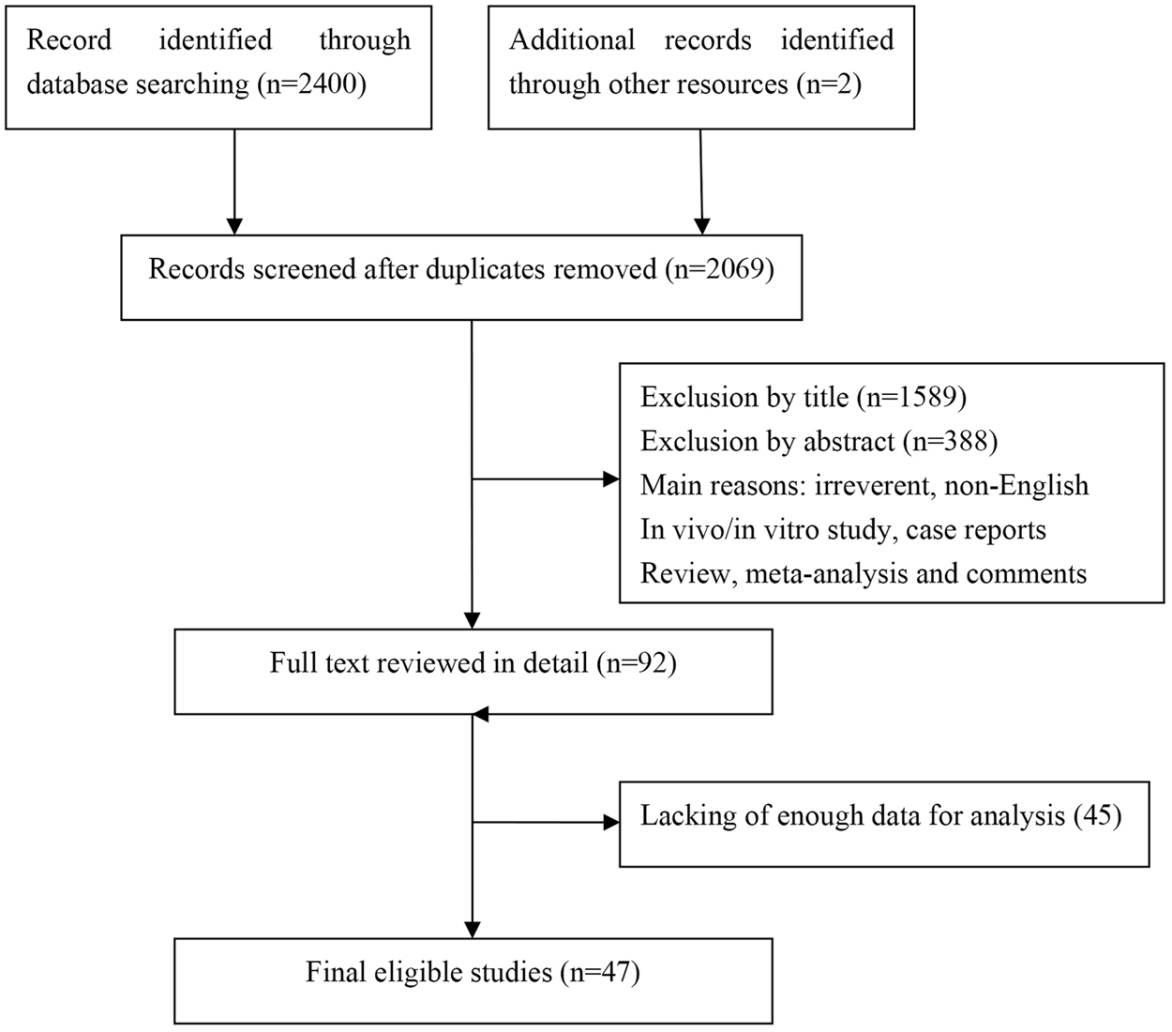
Flow chart of study selection.

The literature review process is shown in Fig. 1. The initial search strategies retrieved a total of 2,402 potentially relevant articles. After screening the titles or abstracts, 1977 studies were excluded as irreverent, non-English, *in vivo*/*in vitro* studies, case reports, reviews, meta-analyses and comments. After reading the full texts of the remaining articles, 45 studies lacking sufficient data for further analysis were discarded. Forty-seven studies with 11,444 patients were finally included for meta-analysis.

Referring to Table 1 for the major characteristics included of the studies. Among the 47 studies, twenty-three investigated PD-L1 expression in NSCLC^6, 19–40^, thirteen in adenocarcinoma (ADC)^41–53^, six in squamous cell carcinoma (SCC)^54–59^, two in small cell lung cancer (SCLC)^60, 61^, and two investigated PD-L1 in pulmonary lymphoepithelioma-like carcinoma (LELC)^62, 63^, and one investigated PD-L1 in pulmonary pleomorphic carcinoma (PPC)^64^. Thirty-seven studies were conducted with Asian patients, and 10 studies were conducted with non-Asians patients. Twenty-three studies included non-metastatic lung cancer patients, while 5 studies involved metastatic disease, and 17 studies involved both non-metastatic and metastatic diseases. The Newcastle–Ottawa Quality Assessment Scale (NOS) scores of the studies ranged from 4 to 8, with a mean value of 6.92.

**Table 1:** Characteristics of the studies included in the meta-analysis.

Author	Year	Country	Tumor type	No. of patients	Stage	Detection method	PD-L1 positive	Outcome	HR estimation	Prognostic value	Quality score
Mu *et al*.	2011	China	NSCLC	109	I-III	IHC	53.2% (58/109)	OS	K-M	Poor	4
Chen *et al*.	2012	China	NSCLC	120	I-III	IHC	57.5% (69/120)	OS	HR	Poor	8
Azuma *et al*.	2014	Japan	NSCLC	164	I-III	IHC	50% (82/164)	OS	HR	Poor	8
Mao *et al*.	2014	China	NSCLC	128	I-III	IHC	72.7% (96/128)	OS	HR	Poor	7
Velcheti *et al*.	2014	Greek	NSCLC	303	I-IV	QIF	24.8 (75/303)	OS	K-M	Good	6
Velcheti *et al*.	2014	USA	NSCLC	155	I-IV	QIF	36.1% (56/155)	OS	HR	Good	6
Cooper *et al*.	2015	Australia	NSCLC	678	I-III	IHC	7.4 (50/678)	OS	HR	Good	6
D’incecco *et al*.	2015	Italy	NSCLC	123	IV	IHC	55.3% (68/123)	OS	K-M	NR	6
Schmidt *et al*.	2015	Germany	NSCLC	321	I-III	IHC	24% (77/321)	OS	HR	Good	7
Tang *et al*.	2015	China	NSCLC	170	IIIB-IV	IHC	65.9% (112/170)	OS	HR	NR	8
Ameratunga *et al*.	2016	Australia	NSCLC	420	I-III	IHC	23.8% (100/420)	OS	HR	NR	7
Chen *et al*.	2016	China	NSCLC	48	I-IV	IHC	64.6% (31/48)	OS	K-M	NR	6
Inoue *et al*.	2016	Japan	NSCLC	654	I-III	IHC	30.7% (201/654)	OS	HR	Poor	7
Ji *et al*.	2016	China	NSCLC	100	I-III	IHC	40% (40/100)	OS	HR	Poor	6
Shimoji *et al*.	2016	Japan	NSCLC	220	I-IV	IHC	31.8% (70/220)	OS	K-M	Good	6
Sorensen *et al*.	2016	USA	NSCLC	204	IV	IHC	75% (153/204)	OS	HR	NR	8
Sun *et al*.	2016	Korea	NSCLC	1070	I-IV	IHC	44.7% (478/1070)	OS	HR	Poor	8
Teng *et al*.	2016	China	NSCLC	126	I	IHC	19.8% (25/126)	OS	HR	NR	7
Tokito *et al*.	2016	Japan	NSCLC	74	III	IHC	74.3% (55/74)	OS	HR	NR	6
Lgawa *et al*.	2017	Japan	NSCLC	229	I-III	IHC	52.4% (120/229)	OS	HR	NR	7
Okita *et al*.	2017	Japan	NSCLC	91	IA-IIIA	IHC	14.3% (13/91)	OS	HR	Poor	7
Takada *et al*.	2017	Japan	NSCLC	499	I-III	IHC	37.9% (189/499)	OS	HR	Poor	6
Tsao *et al*.	2017	Canada	NSCLC	982	I-IV	IHC	32% (314/982)	OS	HR	NR	8
Zhou *et al*.	2017	China	NSCLC	108	I-IV	IHC	40.7% (44/108)	OS	HR	Poor	7
Yang *et al*.	2014	China	ADC	163	I	IHC	39.9% (65/163)	OS	K-M	NR	8
Zhang *et al*.	2014	China	ADC	143	I-III	IHC	49% (70/143)	OS	K-M	Poor	7
Lin *et al*.	2015	China	ADC	56	IV	IHC	53.6% (30/56)	OS	HR	Good	8
Cha *et al*.	2016	Korea	ADC	323	I-IV	IHC	18.6% (60/323)	OS	HR	Poor	6
Huynh *et al*.	2016	USA	ADC	261	I-IV	IHC	36.5% (95/261)	OS	K-M	Poor	6
Lnamura *et al*.	2016	Japan	ADC	268	I-IV	IHC	16% (43/268)	OS	HR	Poor	7
Song *et al*.	2016	China	ADC	385	I-III	IHC	48.3% (186/385)	OS	HR	NR	7
Takada *et al*.	2016	Japan	ADC	417	I-III	IHC	20.4% (85/417)	OS	HR	Poor	7
Hirai *et al*.	2017	Japan	ADC	94	I	IHC	16% (15/94)	OS	HR	Poor	8
Mori *et al*.	2017	Japan	ADC	296	NR	IHC	36.1% (107/296)	OS	HR	Poor	7
Toyokawa *et al*.	2017	Japan	ADC	292	I	IHC	16.1% (47/292)	OS	K-M	Poor	6
Uruga *et al*.	2017	USA	ADC	109	II-III	IHC	51.4% (56/109)	OS	K-M	NR	6
Wu *et al*.	2017	China	ADC	133	I-IV	IHC	13.5% (18/133)	OS	HR	Poor	8
Kim *et al*.	2015	Korea	SCC	331	I-III	IHC	26.9% (89/331)	OS	K-M	NR	4
Ilie *et al*.	2016	France	SCC	56	I-IV	IHC	82.1% (46/56)	OS	K-M	NR	7
Yang *et al*.	2016	China	SCC	105	I	IHC	56.2% (59/105)	OS	HR	Good	8
Guo *et al*.	2017	China	SCC	128	III-IV	IHC	61.7% (79/128)	OS	K-M	Poor	7
Takada *et al*.	2017	Japan	SCC	205	NR	IHC	51.7% (106/205)	OS	HR	NR	7
Zhang *et al*.	2017	China	SCC	84	I-III	IHC	58.3% (49/84)	OS	HR	Poor	7
Ishii *et al*.	2015	Japan	SCLC	102	I-IV	IHC	71.6% (73/102)	OS	HR	Good	8
Miao *et al*.	2016	China	SCLC	83	I-IV	IHC	51.8% (43/83)	OS	HR	Poor	8
Jiang *et al*.	2015	China	LELC	79	I-IV	IHC	63.3% (50/79)	OS	HR	NR	8
Fang *et al*.	2015	China	LELC	113	I-IV	IHC	74.3% (84/113)	OS	HR	NR	7
Chang *et al*.	2016	China	PPC	122	I-IV	IHC	70.5% (86/122)	OS	HR	Poor	8

Abbreviations: NSCLC = non small cell lung cancer, ADC = adenocarcinoma, SCC = squamous cell carcinoma, SCLC = small cell lung cancer, LELC = pulmonary lymphoepithelioma-like carcinoma, PPC = pulmonary pleomorphic carcinoma, IHC = immunohistochemistry, QIF = quantitative fluorescence, OS = overall survival, HR = hazard ratio, K-M = Kaplan–Meier curve, NR = not revelant.

### Correlation of PD-L1 expression with prognosis

**Figure 2:**
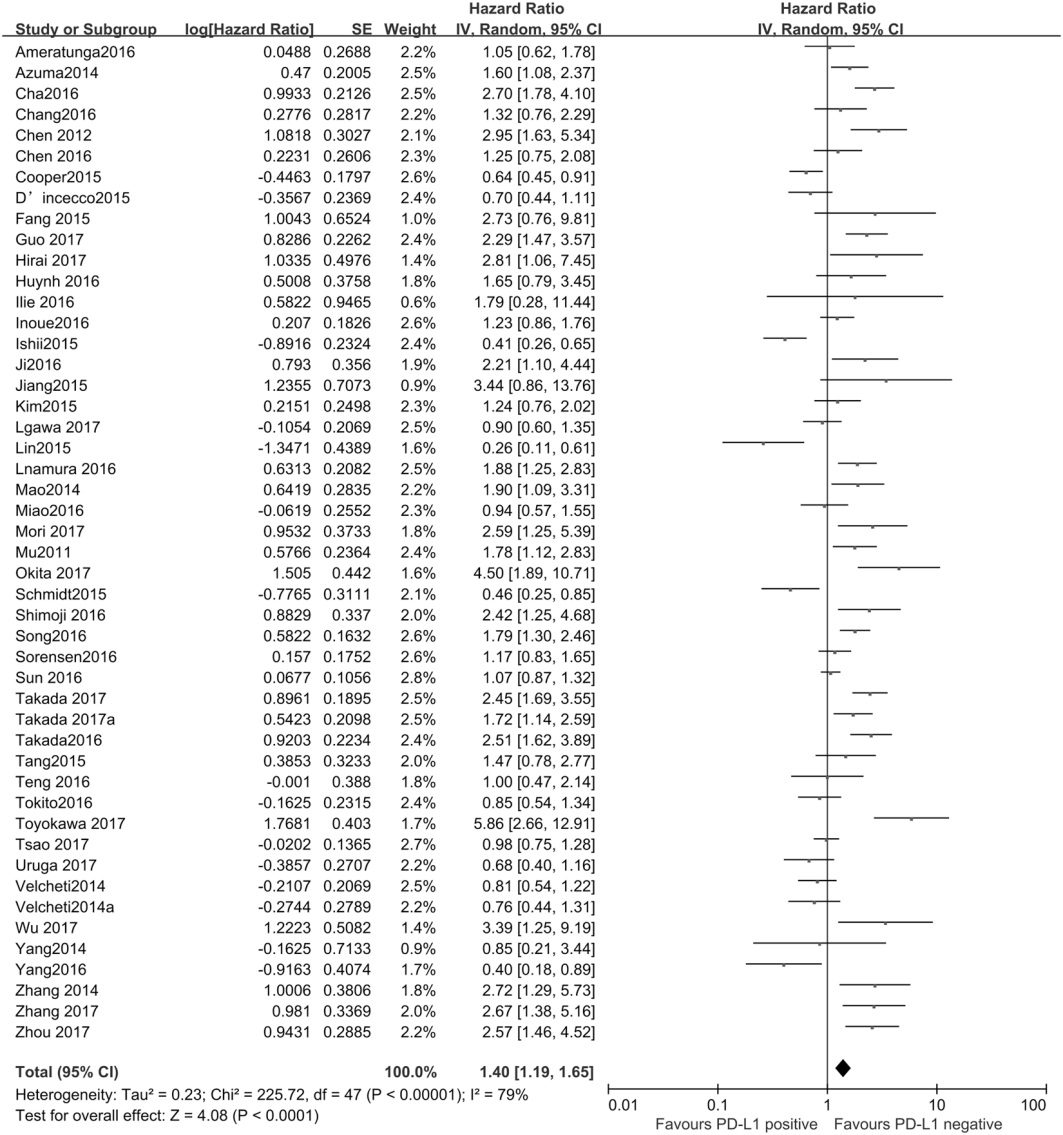
Forest plot describing the association between PD-L1 expression and OS of patients with lung cancer.

The correlation between the expression of PD-L1 and overall survival (OS) in lung cancer is shown in Fig. 2. The meta-analysis indicated that PD-L1 expression is a correlative factor of OS, with the pooled hazard ratio (HR) values of 1.40 (95% CI: 1.19–1.65, *P* < 0.001) for OS using a random model with significant heterogeneity (I^2^ = 79%, *P* < 0.001).

**Figure 3:**
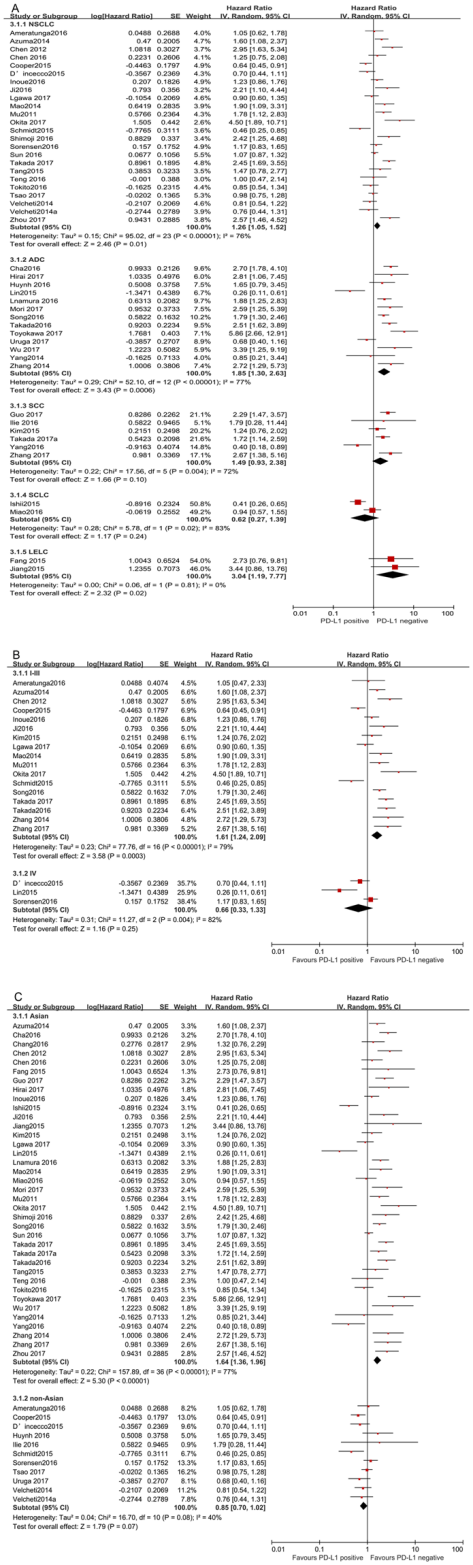
Forest plot describing subgroup analysis of the association between PD-L1 expression and OS. (**A**) histological types, (**B**) TNM stage, (**C**) ethnicity.

To explore the sources of potential heterogeneity, subgroup analysis for OS was conducted according to histology type, TNM stage and ethnicity. Subgroup analyses based on histology types showed that PD-L1 expression significantly reduced the OS of NSCLC patients (HR = 1.26, 95% CI: 1.05–1.52, *P* = 0.01) and LELC patients (HR = 3.04, 95% CI: 1.19–7.77, *P* = 0.02), but not SCLC (HR = 0.62, 95% CI: 0.27–1.39, *P* = 0.24).To further examine the effects of different subtypes of NSCLC on survival, a subgroup analysis was conducted in patients with ADC and SCC. The results revealed that increased PD-L1 expression was associated with poor prognosis in patients with ADC (HR = 1.85, 95% CI: 1.30–2.63, *P* < 0.001), but not in SCC (HR = 1.49, 95% CI: 0.93–2.38, *P* = 0.10). In addition, subgroup analyses according to TNM stage showed that increased PD-L1 expression impacted OS negatively for lung cancer patients in stage I-III (HR = 1.61, 95% CI: 1.24–2.09, *P* < 0.001), but not in stage IV (HR = 0.66, 95% CI: 0.33–1.33, *P* = 0.25). When grouped according to ethnicity, the combined HRs of Asian studies and non-Asian studies were 1.64 (95% CI: 1.36–1.96, *P* < 0.001) and 0.85 (95% CI: 0.70–1.02, *P* = 0.07), respectively, indicating that PD-L1 is an indicator of the poor prognosis in Asian populations, but not in non-Asian populations (Fig. 3).

### Correlation of PD-L1 with clinicopathological features

The correlation between PD-L1 expression and clinicopathological parameters of lung cancer is shown in Supplementary Figs 1–7. The pooled results showed that PD-L1 expression was increased in male (OR = 1.46, 95%CI: 1.24–1.71, *P* < 0.001), smoker (OR = 1.57, 95% CI: 1.28–1.93, *P* < 0.001), patients with SCC (OR = 1.59, 95% CI: 1.11–2.26, *P* = 0.01), a higher histological grade (OR = 2.55, 95% CI: 2.05–3.19, P < 0.001), larger tumor sizes (OR = 1.70, 95% CI: 1.29–2.25, *P* < 0.001), positive lymph nodal metastasis (OR = 1.34, 95% CI: 1.19–1.50, *P* < 0.001) and TNM stage (OR = 1.45, 95% CI: 1.18–1.78; *P* < 0.001). The analysis of the relation of PD-L1 expression to histological grade (*P* = 0.07; I^2^ = 39%), tumour size (*P* = 0.25; I^2^ = 24%), and lymph nodal metastasis status (*P* = 0.02; I^2^ = 42%) presented no heterogeneity; thus, a fixed effect model was used. The other analyses above were performed using the random effects model.

### Correlation of PD-L1 with major driver mutations

**Figure 4:**
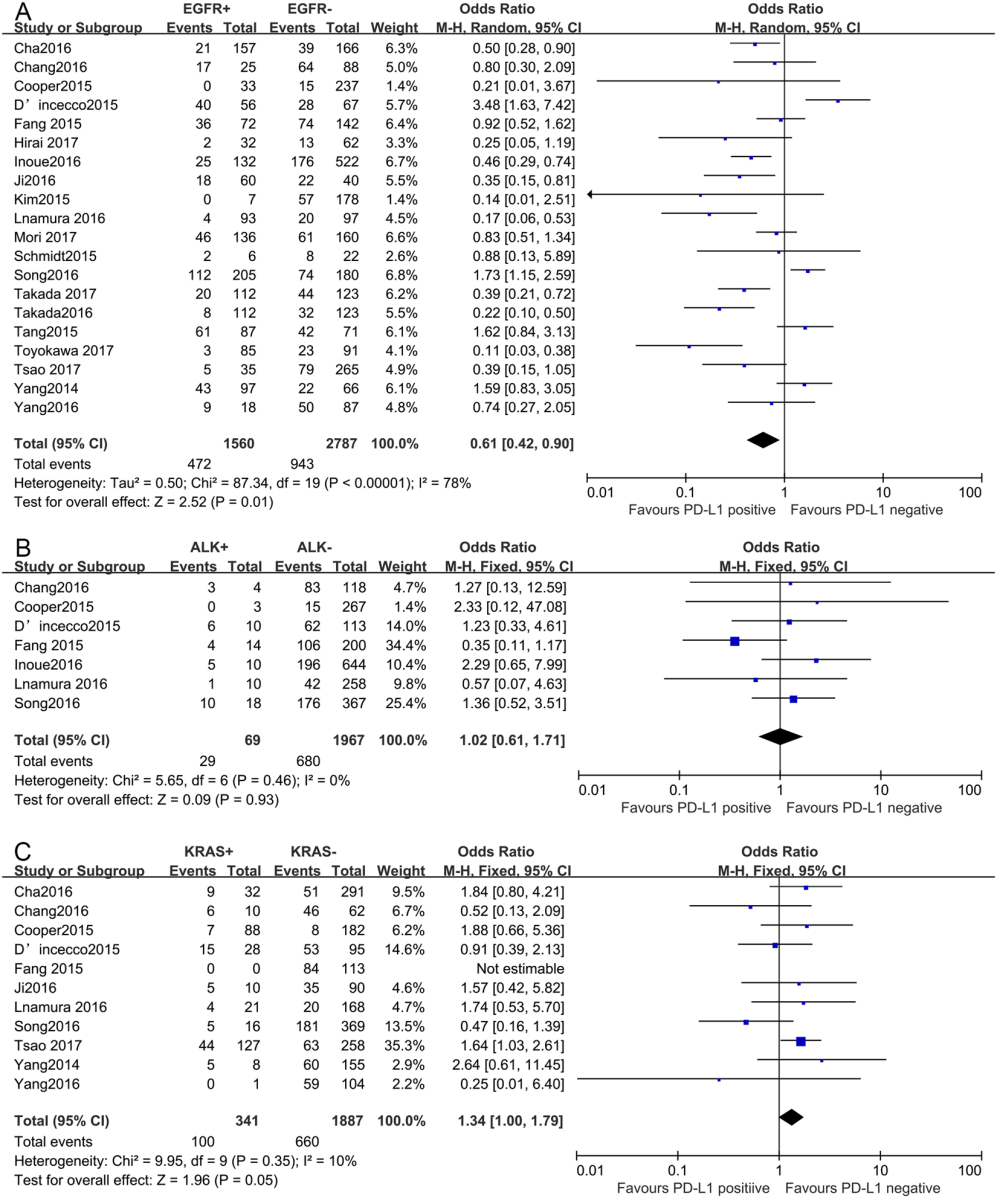
Forest plots for the association between PD-L1 expression and major drive mutations. (**A**) EGFR status, (**B**) ALK status, (**C**) KRAS status.

To further understand the role of PD-L1 expression as a biological marker, we investigated the relevance of increased PD-L1 expression and major driver mutations (EGFR/ALK/KRAS). As shown in Fig. 4, PD-L1 expression was associated with EGFR wild-type status (OR = 0.61, 95% CI: 0.42–0.90, *P* = 0.01), while no associations were identified between PD-L1 expression and ALK rearrangements (OR = 1.02, 95% CI: 0.61–1.71, *P* = 0.93) or KRAS mutations (OR = 1.34, 95% CI: 1.00–1.79, *P* = 0.05).

### Publication bias and sensitivity analysis

**Figure 5:**
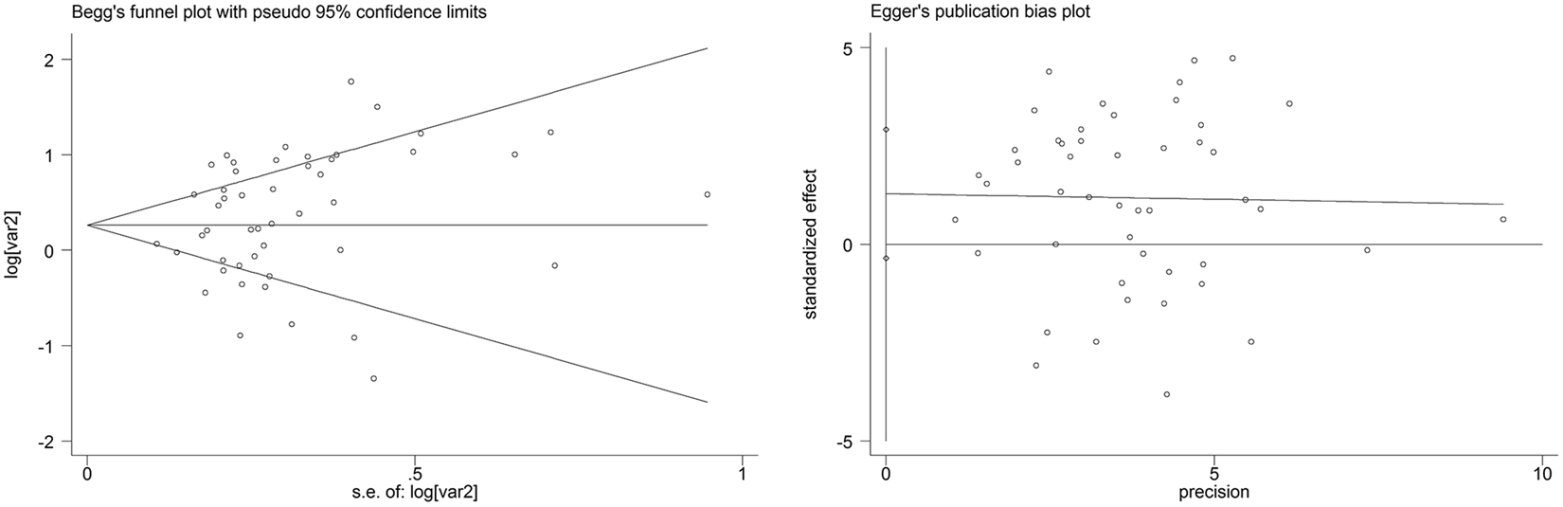
(**A**) Begg’s funnel plot with 95% confidence intervals for OS publication bias testing, (**B**) Egger’s funnel plot with 95% confidence intervals for OS publication bias testing.

Begg’s and Egger’s test were performed to evaluate the publication bias in the literature. And no indicator of publication bias among these studies was present. The *P* values for these tests were 0.237 and 0.120, respectively (Fig. 5). (Statistical significance was set at P < 0.05). Meanwhile, the sensitivity analysis was performed to assess the stability of the present meta-analysis by omitting one study. The results demonstrated that none of the studies influenced the overall HRs, suggesting that the results of the study are credible.

## Discussion

High PD-L1 expression has been observed in various solid tumours, and a previous study demonstrated that the expression of PD-L1 contributes to poor prognosis^65^. Although heavily investigated; it remains controversial for the prognostic value of PD-L1 expression in lung cancer, reflecting the inconsistent results of previous studies. This meta-analysis included 47 studies with 11,444 patients to evaluate the significance of increased PD-L1 to the prognosis of lung cancer. The results of the present analysis showed increased PD-L1 expression was associated with poor prognosis in lung cancer patients.

According to subgroup analysis, high PD-L1 expression was an indicator of poor prognosis in Asian populations, but not in non-Asian populations, suggesting that the association between PD-L1 expression and prognosis is dependent on ethnicity. Different histological types of lung cancer process different biological characteristics. To reduce the heterogeneity of study, we performed a subgroup analysis on the basis of different histological types. The pooled results demonstrated that increased PD-L1 expression was an adverse prognostic factor for NSCLC and LELC, but not for SCLC. Our study analyzed the relationship between PD-L1 expression and prognoses of LELC and SCLC for the first time. This study provides important evidence on the prognostic value of the PD-L1 expression in LELC and SCLC patients. A potential correlation between PD-L1 expression and OS of patients with NSCLC was evaluated in previous meta-analyses^66–69^. The results of three meta-analyses revealed that NSCLC patients with increased PD-L1 expression had a poor OS^66–68^. Another meta-analysis did not indicate PD-L1 as a prognostic predictor for NSCLC^69^. However, the combined sample size of the four meta-analyses was relatively small. In addition, the four meta-analyses did not include SCLC and LELC, nor the investigation of the association between increased PD-L1 expression and driver mutations. Compared with those meta-analyses, more studies have been included in our research. Different thresholds to define positivity expression and particularly different baseline characteristics hinder the comparison of different studies reporting correlation of PD-L1 expression with OS in NSCLC. Standardized methods and definitions of PD-L1 positivity are clearly needed to facilitate studies of PD-L1 as a prognostic biomarker. Thus, a large multicenter study using the same antibody and cutoff of PD-L1 expression may be helpful to obtain more accurate results.

Several clinical trials using anti-PD-1 and anti-PD-L1 monoclonal antibodies, including nivolumab (BMS-936558)^70, 71^, pembrolizumab (MK-3475)^72^, and atezolizumab (MPDL3280A)^73^ have shown promising clinical activity in advanced NSCLC. In the era of precision medicine, it is particularly important to screen patients who are most likely to benefit from PD-1/PD-L1 antibody immunotherapy. Preliminary results suggested that high PD-L1 expression was associated with higher clinical activity of anti PD-1/PD-L1 monoclonal antibodies^74^. Therefore, the identification of patients with high PD-L1 expression is a vital question for anti-PD-1/PD-L1 therapy. In the present study, we investigated the relation of PD-L1 expression to clinicopathological factors. According to the pooled analysis, the expression of PD-L1 was increased in male, smoker, patients with SCC, a higher histological grade, larger tumour size, positive lymph nodal metastasis, and later clinical stage. These patients might benefit more from treatment targeting the PD-1/PD-L1 pathway. These data suggest that increased PD-L1 expression might promote lung cancer invasion and metastasis, leading to the poor prognosis of patients with lung cancer. It has been reported in several studies regarding the association of smoking status with PD-L1 expression in patients with lung cancer. Some studies have shown that the expression of PD-L1 was significantly higher in smokers^28, 46^, whereas other studies could not confirm this finding^23, 24, 45, 54^. In the present study, patients with high PD-L1 expression were associated with smoking status in lung cancer patients.

Accumulating evidence revealed the relationship between PD-L1 expression and driver mutations. EGFR mutations represent one of the most frequent driver mutations in NSCLC, particularly in ADC. Previous studies revealed that activating EGFR mutations induced PD-L1 expression in EGFR-driven NSCLC in cell lines and an animal model^21, 75^. Moreover, as observed in NSCLC cell lines, there was a high level of PD-L1 expression in NSCLC patients harboring EGFR mutations^21, 24, 45^. However, some studies have shown that PD-L1 positivity was more frequent in EGFR wild-type^28, 44, 46^, and other studies have shown no association between PD-L1 expression and EGFR mutations^23, 26, 27^.The present meta-analysis investigated the correlation of PD-L1 expression with EGFR mutations in lung cancers. The results of the present study showed that high PD-L1 expression was associated with EGFR mutations. The discrepancies among different studies might reflect the heterogeneous study population and variable definitions of PD-L1 expression. Additional studies are needed to further analyze this issue. In addition, we showed that increased PD-L1 is not associated with ALK rearrangements and KRAS mutations.

There are several limitations of the present study that should be acknowledged. First, the sample size of SCLC and LELC studies included in the present meta-analysis was relatively small; therefore, the pooled data might be less than the statistical power. Hence, additional well-designed studies with larger sample sizes are needed to provide a more comprehensive evaluation of the prognostic value of PD-L1 expression in patients with SCLC and LELC. Second, the HR values of some studies were extracted from survival curves, which is less reliable than direct data provided in the original literature. Third, the distinct antibodies and different cut-off levels of PD-L1 expression among diverse studies might also impact the accuracy of prognostic estimation for lung cancer. Moreover, some inevitable publication bias might exist in the present meta-analysis, as many negative studies could not be published. Furthermore, significant heterogeneity existed in the results, although we calculated the pooled subgroup data using random-effects models. The observed heterogeneity might reflect differences based on different baseline characteristics, study designs, or treatment protocols. Finally, all of included studies were retrospectively collected, which might have introduced heterogeneity from variable treatments.

In conclusion, despite the limitations described above, this study presents the first meta-analysis to systematically assess the association of PD-L1 expression with lung cancer survival and driver mutations. The results demonstrated that high PD-L1 expression represents an unfavorable biomarker in LELC and NSCLC, but not in SCLC. In addition, increased PD-L1 expression is correlated with EGFR wild-type status. To strengthen these findings, the validation of the prognostic value of PD-L1 expression in patients with lung cancer requires further studies.

## Methods

This meta-analysis was performed according to the preferred reporting items for systematic reviews and meta-analysis (PRISMA) statement^76^. The present study was based on data from previously published studies, and therefore, ethical approval was not required.

### Literature search

We conducted a systematic literature search for published articles in the PubMed, EMBASE, and Cochrane databases from January 1999 to July 2017. The search terms included the following keywords: (PD-L1 OR B7-H1 OR CD274 OR programmed cell death 1 ligand 1 protein) AND (lung cancer OR lung neoplasms OR pulmonary cancers). Furthermore, we manually searched the abstracts of the annual meetings of American Society of Clinical Oncology (ASCO), European Society of Medical Oncology c (ESMO) and the World Conference of Lung Cancer (WCLC) from 1999 to 2017. To explore additional studies, we also reviewed the reference lists of relevant articles.

### Eligibility criteria

The following inclusion criteria were used: (1) All patients were histologically confirmed as having lung cancer; (2) PD-L1 expression was detected by immunohistochemistry (IHC) or quantitative immunofluorescence (QIF) in primary NSCLC tissue; (3) Studies showed a correlation between PD-L1 expression and overall survival; (4) Studies showed a correlation between PD-L1 expression and clinicopathological features; (5) Studies provided sufficient information to extract the HR and 95% CI date for OS; and (6) articles were published in English. Studies that did not meet the inclusion criteria were excluded. When several studies were conducted using the same cohort of patients, only the most recent study was included.

### Data extraction

Two authors (ZMH and LGL) independently conducted the data extraction, and a third reviewer (WY) resolved any discrepancies. The following information was extracted: name of the author, year of publication, country, tumour type, number of patients, stage, detection method, PD-L1-positive expression, outcome, clinicopathological parameters and HRs and 95% CIs for OS. When the HR values were not directly reported, we obtained the additional data from the original authors. And the data was extracted from survival curves using the methods of Parmar under the circumstances of no response^77^. Two reviewers (ZS and WY) independently conducted the quality assessment for each study using the NOS, and any discrepancies were resolved after revisiting the original study and discussion until consensus was reached. The NOS maximum possible score was 9 points, and studies that receiving a score of 6 or higher were considered high quality^78^.

### Statistical methods

The HR and its 95% CI values were used to evaluate the association between PD-L1 expression and survival, and the pooled OR and 95% CI values were used to determine the relationship between PD-L1 expression and clinicopathological features. Statistical heterogeneity between studies was assessed using the chi-squared test and I^2^. A P value <0.1 orI^2^ values of >50% were indicative of significant heterogeneity, with a random effects model being used; a fixed effects model otherwise. A subgroup analysis was conducted to explore the potential heterogeneity among studies. Potential publication bias was assessed using Egger’s and Begg’s tests. The meta-analysis was performed using Review Manager 5.3 (Revman the Cochrane Collaboration; Oxford, England) and STATA version12.0 (Stata Corporation; College Station, TX, USA). All statistical analyses were 2-sided, and P values < 0.05 were defined as statistically significant.

## Electronic supplementary material

Supplementary Fig.1-7
