# Supplementary material for: PD-L1 expression in lung cancer and its correlation with driver mutations: a meta-analysis
Source: Sci Rep. 2017 Aug 31;7:10255. doi: 10.1038/s41598-017-10925-7 (PMC5578960; doi:10.1038/s41598-017-10925-7)

**Article title**: **PD-L1 expression in lung cancer and its correlation with driver mutations: a meta-analysis**

**Author names and affiliation**: Minghui Zhang ^1*^, Guoliang Li^2*^, Yanbo Wang ^3^, Yan Wang^4^, Shu Zhao^1^, Haihong, Pu^1^, Hongli Zhao^1^, Yan Wang ^1^

***Correspondence to:**Yan Wang, **e-mail:** wangyan11lou@163.com

+these authors contributed equally to this work

**Supplementary Fig.1-7. Forest plots for the association between PD-L1 expression and clinicopathological features. (1) gender, (2) smoking status, (3) histology types, (4) histological grade, (5) tumor size, , (6) lymph node metastasis, (7) TNM stage.**

Figure 1


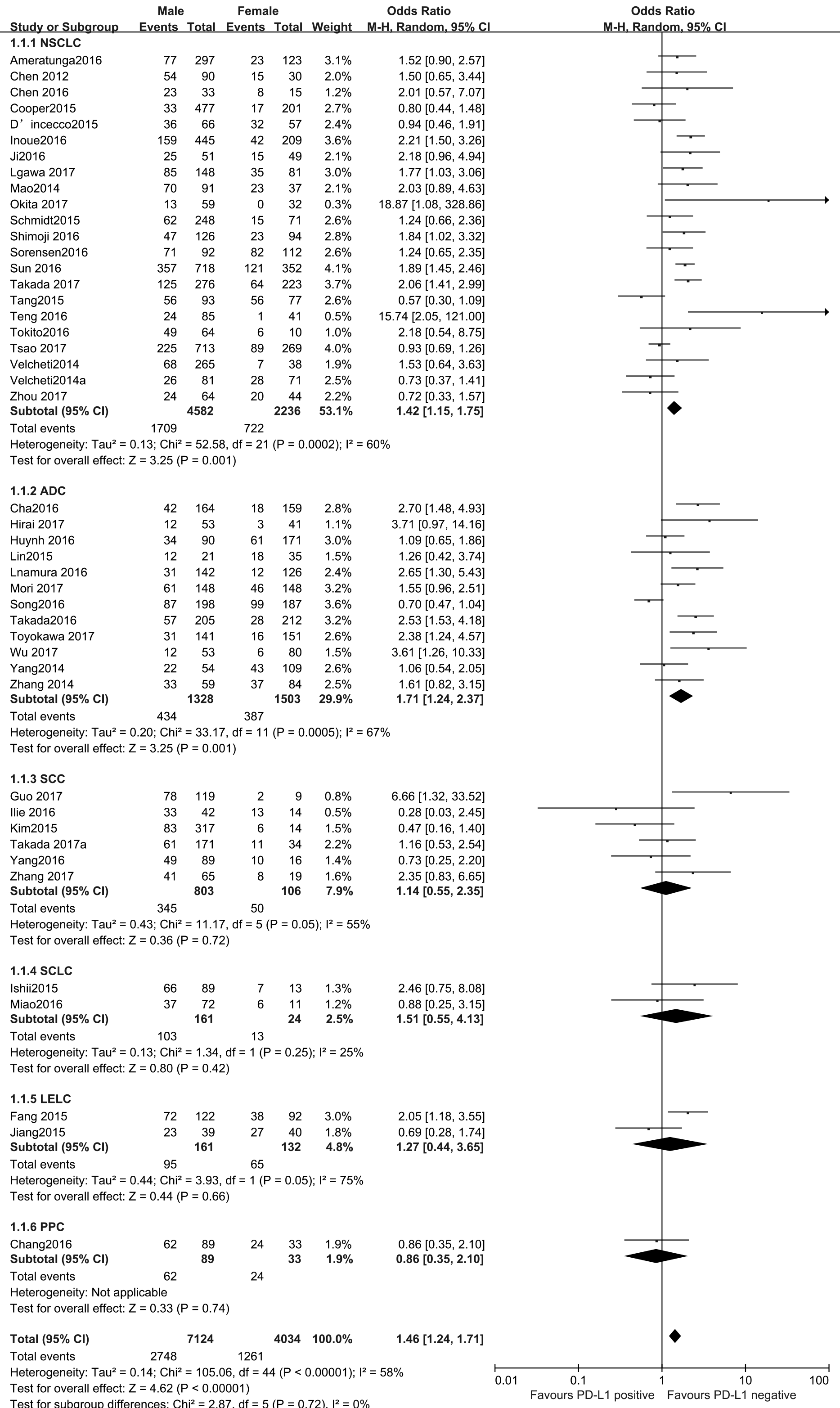


Figure 2


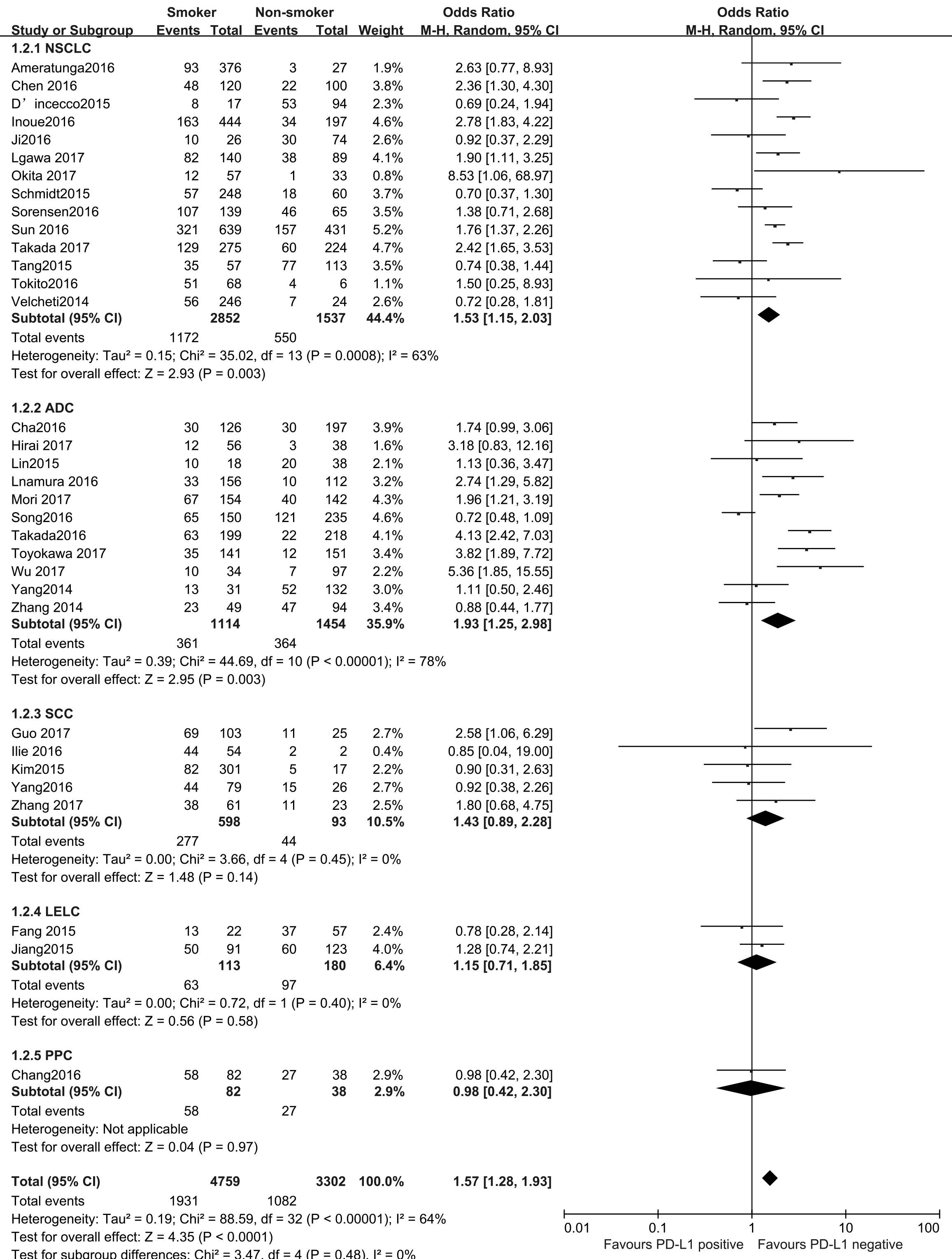


Figure 3


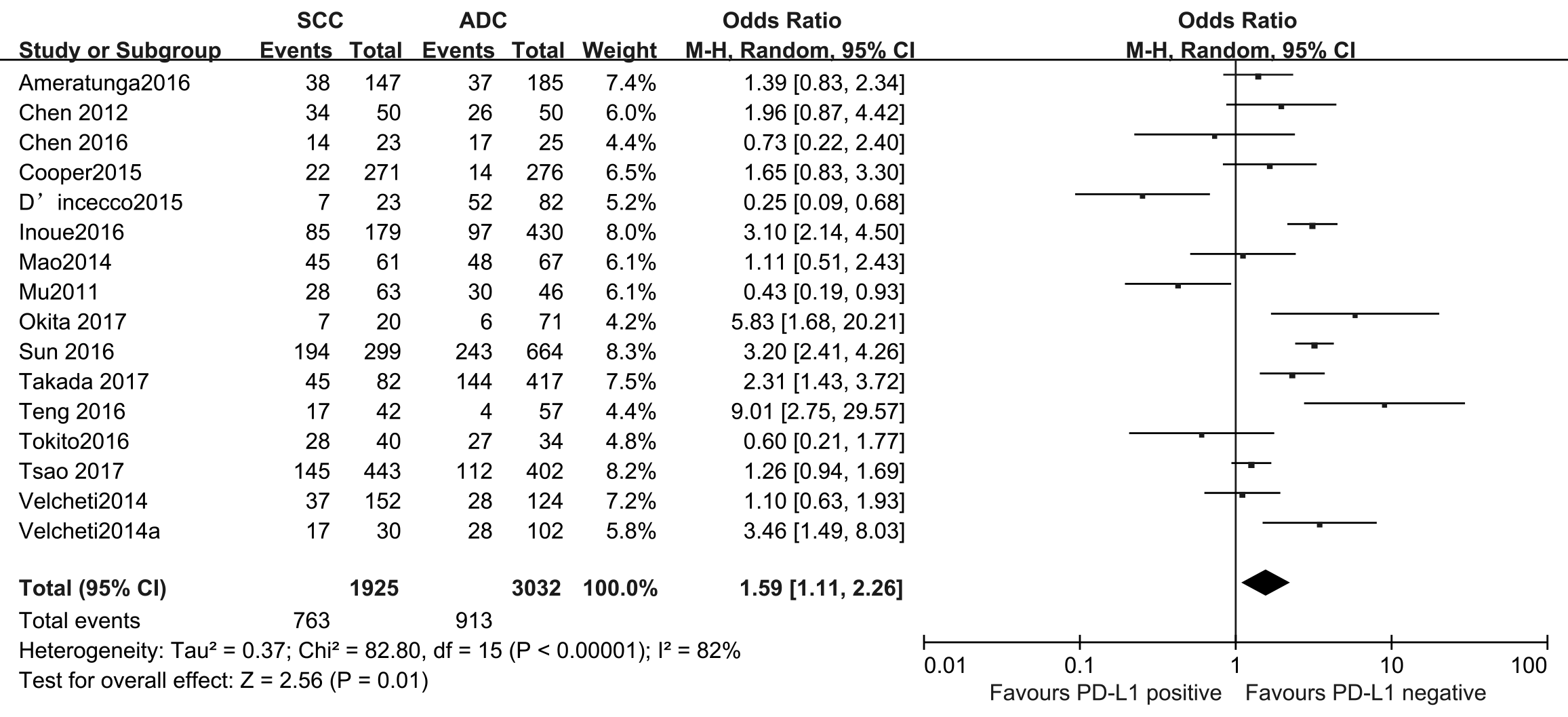


Figure 4


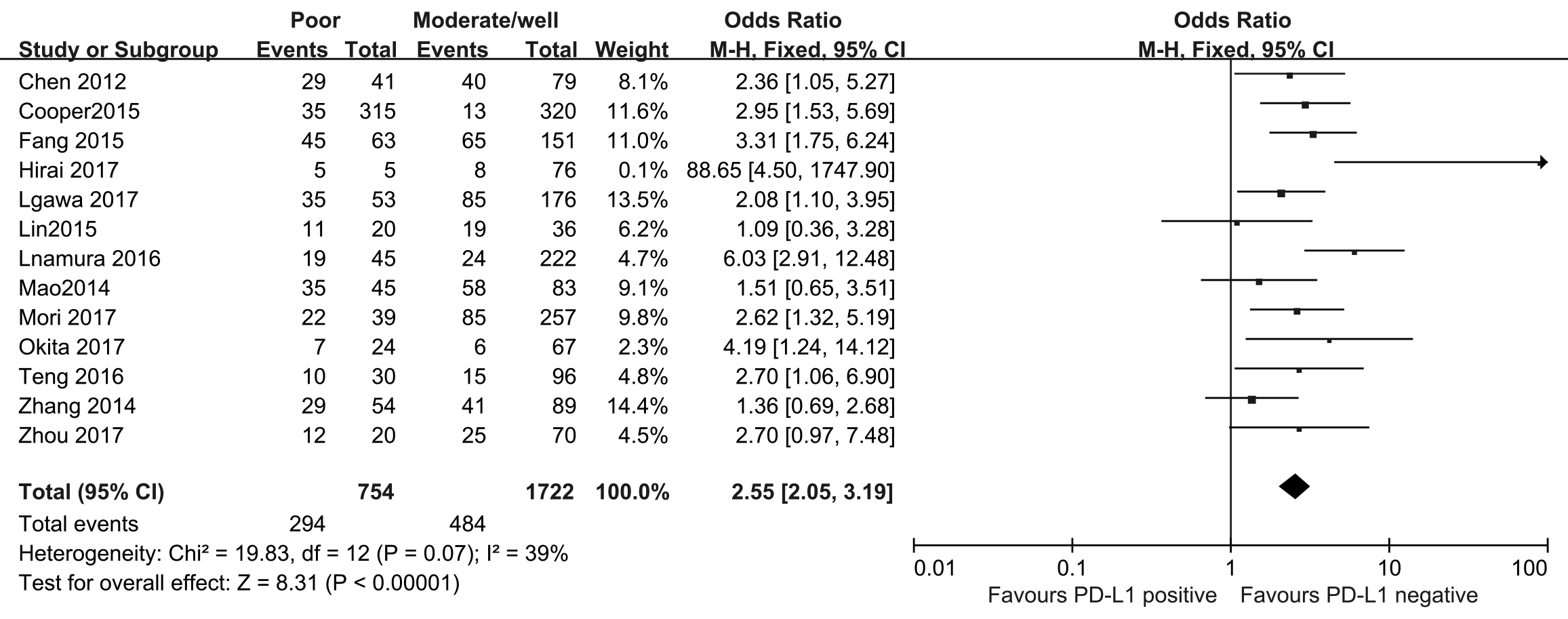


Figure 5


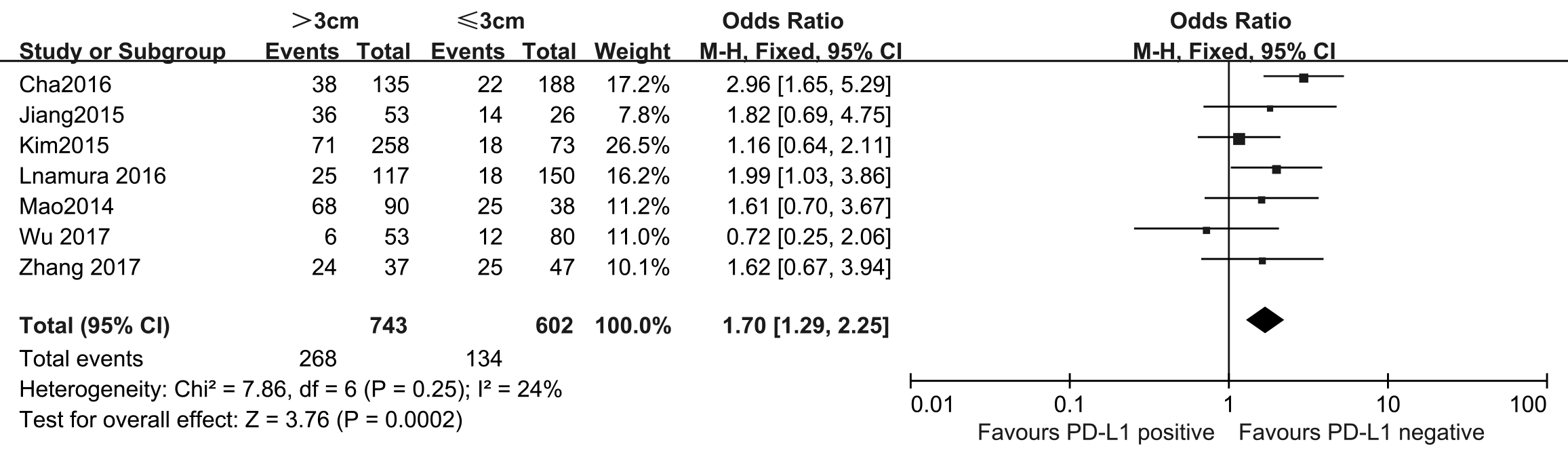


Figure 6


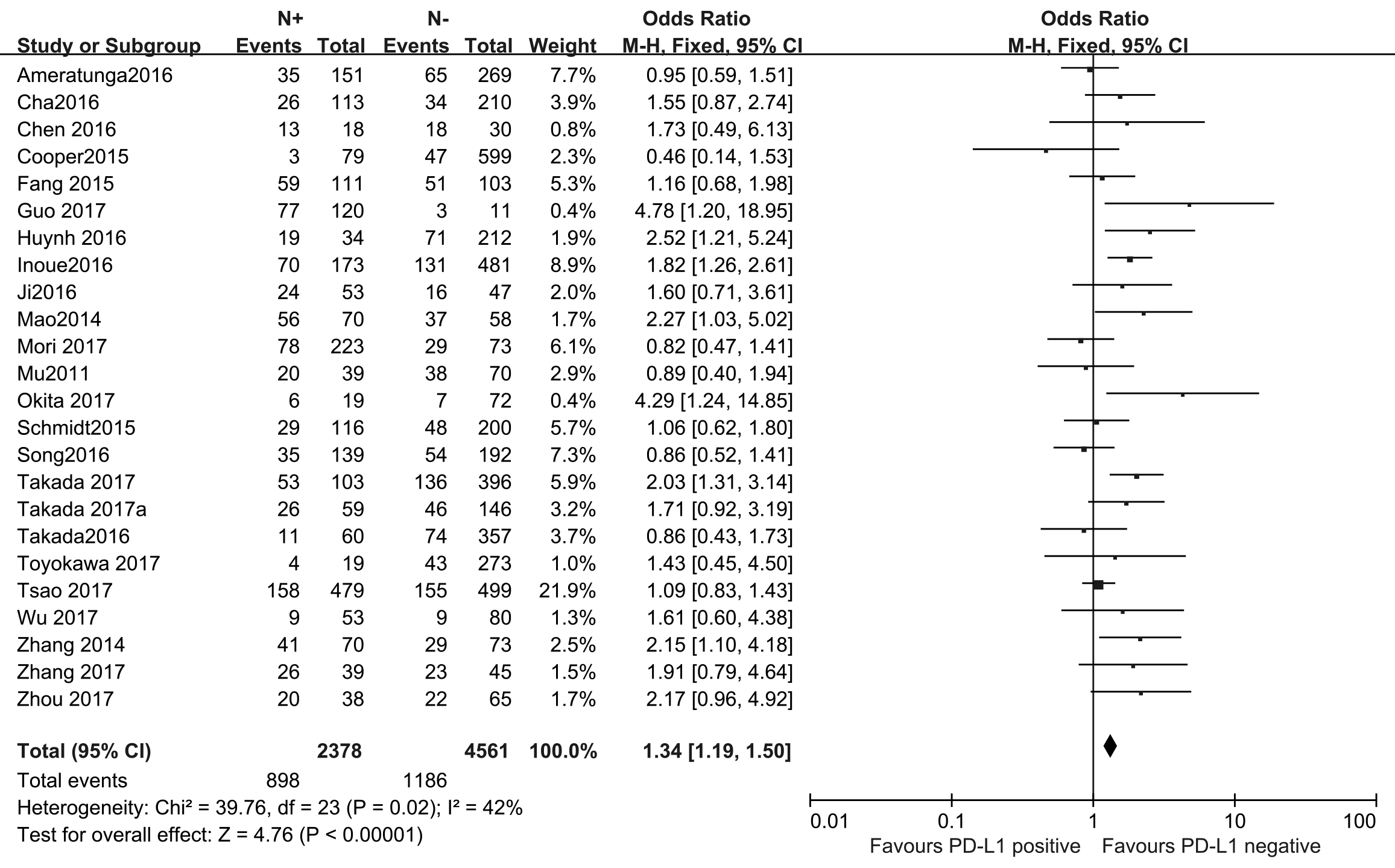


Figure 7


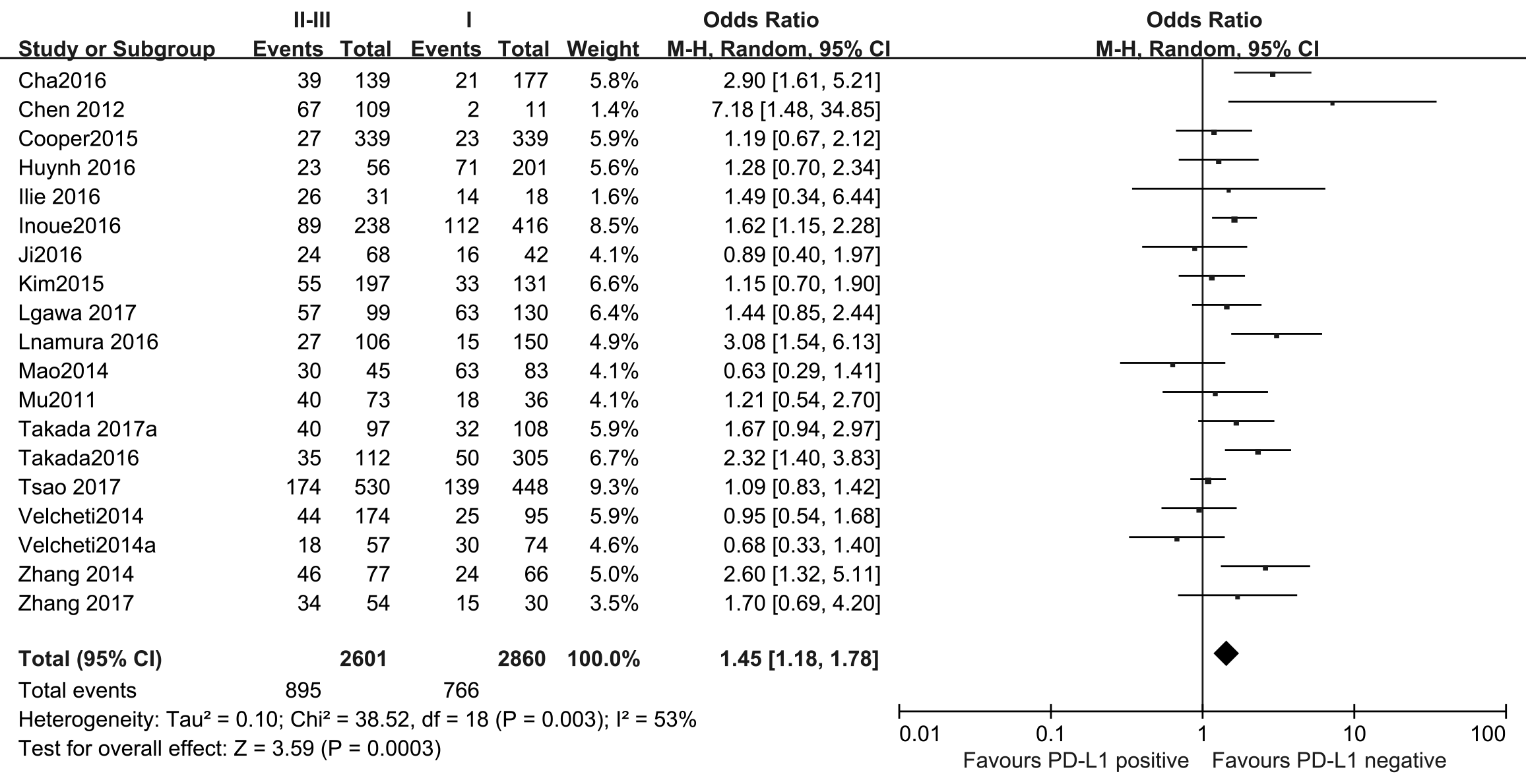

Supplement: Supplementary file 1 — Supplementary Fig.1-7 [file 41598_2017_10925_MOESM1_ESM.doc]
